# Knowledge, attitudes and practices of cervical cancer screening among rural women in KwaZulu-Natal, South Africa

**DOI:** 10.11604/pamj.2022.42.188.26172

**Published:** 2022-07-07

**Authors:** Oluwatosin Omoyeni, Joyce Tsoka-Gwegweni

**Affiliations:** 1 Department of Public Health Medicine, School of Nursing and Public Health, College of Health Sciences, University of KwaZulu-Natal, KwaZulu-Natal, South Africa,; 2 Office of the Dean, Faculty of Health Sciences, University of the Free State, Bloemfontein, South Africa

**Keywords:** Cervical cancer screening, knowledge, attitudes, practices, South Africa, KwaZulu-Natal, rural women

## Abstract

**Introduction:**

cervical cancer continues to be a major cause of morbidity and mortality among women in the developing world. Despite the national cervical cancer screening programme, findings show low levels of knowledge and practices of cervical cancer screening among rural women in South Africa (SA). The purpose of this study was to determine the knowledge, attitudes and practices of cervical cancer screening among rural women in KwaZulu-Natal, SA.

**Methods:**

an observational cross-sectional study was performed. The study was conducted at three rural clinics. A systematic sampling technique was used to select 283 women, aged 18-65 years. Data were collected using a standardised structured self-administered questionnaire. Data were analysed using descriptive and analytic statistics.

**Results:**

findings showed a high level of awareness of cervical cancer (93.3%, n=264) and the Pap smear test (95.1%, n=269). Knowledge of cervical cancer-associated factors, symptoms, screening methods and treatment was poor (28.0%, n=79). An overwhelming majority (81.8%, n=231) displayed a positive attitude towards cervical cancer screening. The practice of cervical cancer screening was fairly good (66.8%, n=189). The results showed that socio-demographics were not statistically significantly associated with knowledge of cervical cancer and cervical cancer screening.

**Conclusion:**

despite the inadequate knowledge of women, attitudes towards cervical cancer and screening were generally positive. Health care practitioners are encouraged to focus not only on creating awareness but also on improving knowledge so as women will not only undergo screening but appreciate the importance of cervical cancer screening.

## Introduction

Cervical cancer is the fourth most common cancer among women across the globe [1-3]. Despite being preventable and curable when detected at an early stage, 570,000 new cases and 311,000 deaths were recorded among women in 2018 [3]. More than 90% of this global mortality occurred in developing countries [1,4]. The incidence of cervical cancer in Africa is on the rise [5,6]. Sub-Saharan Africa is the region with the highest incidence and mortality of cervical cancer in the world, with 75,000 annual new cases and 50,000 annual deaths [7]. These figures contrast with the United States where the annual number of new cases and deaths are 12,990 and 41,200 women, respectively [8]. The most affected regions are Eastern Africa, Middle Africa, Southern Africa, and Melanesia with age-standardised incidence rates (ASIRs) greater than 30 per 100,000 persons [9]. The lowest rates were found in Australia, New Zealand and Western Asia with ASIRs of 5.5 per 100, 000 persons [9]. This marked discrepancy highlights the global inequality in cervical cancer incidence and mortality [10]. This global inequality in developed and developing countries has been linked to divergences in human development, social inequality, preventive health behaviour, living standards and access to effective screening services [11,12]. The extent of cervical cancer in sub-Saharan Africa has received little recognition and prioritisation [13]. This is due to a lack of good quality epidemiological data, lack of national cancer prevention programmes and lack of cancer registries [13].

Cervical cancer is the second most commonly reported cancer among all women in South Africa (SA) [5,9]. Women from poor communities are mostly affected [14]. The high burden of cervical cancer in SA is also fuelled by the high prevalence of HIV and AIDS [15]. Human papillomavirus (HPV) has been established as a necessary cause of nearly all cervical cancer [9,15,16]. Cervical cancer can be prevented at the primary, secondary or tertiary stage [17]. Secondary prevention by screening remains the most effective control strategy of cervical cancer [18]. In developed countries, organised cytology-based screening programmes using Papanicolaou (Pap) smears with coverage of high-risk groups have resulted in a significant reduction of cervical cancer incidence and mortality [15,16]. It has effectively reduced the incidence of cervical cancer by 75-90% in developed countries [19], however, screening in developing countries remains underutilised [20]. Cervical cancer screening coverage in SA is still at 20% nationally for women over the age of 30 years. This is much lower than the Department of Health´s goal of screening at least 70% of women nationally [15,21]. Lack of knowledge about cervical cancer screening has been highlighted as one of the major challenges affecting cervical cancer screening programmes [22]. In a quest to increase cervical cancer screening uptake, i.e. the practice of cervical cancer screening, among women in rural areas, this study aimed to assess the knowledge, attitudes and practices of cervical cancer screening among women at the Valley of Thousand Hills in KwaZulu-Natal, SA.

## Methods

**Study design and setting:** this was an observational, cross-sectional study design with a descriptive and analytic component. The study was conducted from November 2018 to January 2019 at three primary health care (PHC) clinics located at the Valley of Thousand Hills, a rural settlement in KwaZulu-Natal, SA. The inhabitants of most villages in this settlement are poor and unemployed. Poor health services, large families, scarce infrastructure, and low or no incomes are some factors that constrain development in this community. HIV and AIDS have placed an unbearable burden on people in the area [23].

**Study population:** the study population included women aged 18-65 years residing in the Valley of Thousand Hills at the time of the study, attending the antenatal clinic, family planning clinic and general outpatient department at the PHC clinics serving the population in the study area. Women who had a history of hysterectomy or were not willing to give written informed consent were excluded from the study. According to the national guidelines, the current target population for cervical cancer screening in SA is women aged 30 years and above. However, cervical cancer has been reported as the second most frequent cancer in women between the ages of 15-44 years. Also, according to the South African guidelines document, screening should end at age 65 or after hysterectomy (only after a previous negative test, with no history of abnormal test results in the previous 10 years and in HIV-negative women) [24,25]. Women from 18-65 years were considered for this study. A sample of 283 participants was considered statistically adequate. A purposive sampling method was used to choose three clinics in the Valley of Thousand Hills. The data collection dates of every clinic were dependent on the availability of health care workers; no sampling method was used. A systematic sampling technique was used to realise a representative sample from the community. Every third patient starting from the first patient in the queue willing to take part in the study (using the lottery method) was selected until the calculated sample size was reached per clinic. Due to the odd number of the sample size, 95 participants were selected from the first clinic while 94 participants each were selected from the second and third clinics.

**Data collection:** a standardised questionnaire was developed from questionnaires that had been used related to the evaluation of the knowledge, attitude and practice of cervical cancer screening. Questionnaires were distributed to eligible participants at the three clinics. Participants were of different education levels, but spoke the same local language. The questionnaire was in both English and Zulu language. Every participant was free to choose the language of her convenience as well as whether she would complete it herself or require assistance. The questions gathered information about socio-demographic characteristics, knowledge about cervical cancer and cervical cancer screening (Pap smear was the only screening method available at the three study sites at the time of data collection), associated factors and prevention method; attitudes, practices towards cervical screening and behavioural profile. All women enrolled in the study provided written informed consent. After being briefed about the purpose of the study and her rights, every participant was asked to sign the informed consent form before the questionnaires were administered. Research assistants were hired for fieldwork and were ready to address any query. Every effort was made to abide by the research ethics of the University.

**Statistical analysis:** data were collected by trained research assistants and entered into a Microsoft Excel database. Data were processed and analysed using SPSS V26. Categorical data were presented using frequency distribution tables. Categorical explanatory variables were cross-tabulated against dichotomous outcomes. Participants knowledge of cervical cancer and cervical cancer screening was measured using 30 questions. A score of 24-30 is considered indicative of a good level of knowledge while a score of 23 and below is rated as a poor level of knowledge. Participants´ attitude toward cervical cancer screening was measured using five questions. A score of 4-5 is indicative of a positive attitude, while a score of 3 and below is indicative of a negative attitude. Univariable and multivariable logistic regression were planned to determine the relationship between socio-demographics and knowledge of cervical cancer screening. The univariable model was to be run first and every significant relationship with a p-value of less than 0.2 was considered for selection of variables in the univariable regression analysis for inclusion into the multivariable regression model. The variables sex, age and education were also considered to be included in the multivariable regression model.

**Ethical consideration:** ethics approval was obtained from the Biomedical Research Ethics Committee (BREC Ref No: BE227/18) and permission to conduct this study was obtained from relevant authorities before the commencement of data collection. Written informed consent was obtained from all participants. Participant confidentiality was maintained as the questionnaire did not record any personal information such as name, address or identity number.

**Pilot study:** a three-day piloting of questionnaires was conducted among 30 women attending the outpatient department of the three clinics before the commencement of the study (10 questionnaires per clinic). This was done to ensure data quality, efficacy and comprehensibility of the questionnaire before the research was fully conducted. Participants used for the pilot study fit the inclusion criteria of the study, but they were not included in the main study. The principal investigator conducted the pilot study. Conducting the pilot study assisted in editing the questionnaire for a better understanding of the participants.

## Results

A total of 283 questionnaires were completed and collected and all were included in the study analysis, giving a response rate of 100%.

**Demographic characteristics of study participants:** Table 1 summarises the demographic characteristics of study participants. More than half (54.8%) of the participants were in the age group 25-44 years and 76.7% were single. The majority of the participants (81.6%) were Christians. The highest percentage (43.5%) of participants had two to three children while 10.2% had no children. About 55.5% had a secondary education, whilst 6.7% had no formal education. Of all the participants, 38.5% were employed and 29.0% were housewives.

**Table 1:** demographic characteristics of study participants (N=283)

Variables and categories	Frequency (n)	Percentage (%)
**Age group**		
18-24	65	23.0
25-44	155	54.8
45-65	63	22.3
**Marital status**		
Single	217	76.7
Married	66	23.3
**Educational status**		
No formal education	19	6.7
Primary education	55	19.4
Secondary education	157	55.5
Tertiary education	52	18.4
**Religion**		
Christian	231	81.6
Muslim	2	0.7
Other	50	17.7
**Parity**		
0	29	10.2
1	90	31.8
2-3	123	43.5
4+	41	14.5
**Occupation**		
Employed	109	38.5
Housewife	82	29.0
Student	26	9.2
Other	66	23.3

**Knowledge of cervical cancer symptoms and associated factors:** the results as presented in Table 2 indicate that 264 (93.3%) of the participants had heard of cervical cancer. Of those, more than 60% indicated that they first heard about cervical cancer from community health workers (40.9%) and from doctors or nurses (24.6%). Participants in the study were asked to identify symptoms associated with cervical cancer. Most of the participants correctly selected abnormal vaginal bleeding (81.4%), increased vaginal discharge (73.1%), pelvic pain (58.3%) and pain during sexual intercourse (53.8%).

**Table 2:** overall knowledge of cervical cancer, knowledge of symptoms and associated factors among study participants (N=283)

Variables and categories	Frequency (n)	Percentage (%)
**Overall knowledge of cervical cancer**		
Yes, knowledgeable	79	28.0
No, not knowledgeable	204	72.0
**Ever heard about cervical cancer**		
Yes	264	93.3
No	19	6.7
**Sources of information about cervical cancer (n=264)**		
Community health workers	108	40.9
Doctor or nurse	65	24.6
Media/News/Internet	60	22.7
Brochures, posters	18	6.8
Family and friends	12	4.5
Religious leaders	1	0.4
**Identified symptoms ^a^ (n=264)**		
Abnormal vaginal bleeding	215	81.4
Increase vaginal discharge	193	73.1
Pelvic pain	154	58.3
Pain during sexual intercourse	142	53.8
**Associated factors ^a^ (n=264)**		
Low immunity	201	76.1
Early age at first sexual intercourse	176	66.7
Multiple sexual partners	173	65.5
Cigarette smoking	163	61.7
Family history of cervical cancer	145	54.9
Infection with HPV	120	45.5
Long-term use of oral contraceptive	94	35.6
More than two children	43	16.3

a Multiple responses to the variable, therefore % are more than 100.

HPV = Human Papillomavirus

**Knowledge of cervical cancer, screening methods and treatment:** the results in Table 3 reveal that participants correctly identified a Pap smear (86.6%), HPV testing (45.2%) and visual inspection with acetic acid (VIA) (34.3%) as appropriate cervical screening methods. Additionally, specific drugs given by hospitals (71.0%), surgery (48.8%), radiotherapy (45.6%) were correctly identified as a mode of treating cervical cancer. The majority of the participants (61.7%) indicated that cervical cancer is preventable, whilst 89.0% revealed that cervical cancer can be cured when detected early.

**Table 3:** knowledge of cervical cancer, screening methods and treatment among study participants (N=283)

Variables and categories	Frequency (n)	Percentage (%)
**Cervical cancer screening methods ^a^**		
Pap smear	245	86.6
HPV testing	128	45.2
VIA	97	34.3
Don´t know	11	3.9
**Mode of treatment ^a^**		
Specific drugs given by hospital	201	71.0
Surgery	138	48.8
Radiotherapy	129	45.6
Herbal remedies	16	5.7
**Do you think cervical cancer is preventable?**		
Yes	264	93.3
No	19	6.7
**Can cervical cancer be cured in its earliest stages?**		
Yes	252	89.0
No	24	8.5
Don´t know	7	2.5

a Multiple responses to the variable, therefore % are more than 100.

HPV = Human Papillomavirus; VIA = Visual inspection with acetic acid

**Knowledge of a Pap smear test:** almost all (95.1%) of the participants in the study had ever heard of a Pap smear (Table 4). Of those who had heard of a Pap smear, nurses (47.2%), schoolteachers (17.5%), and gynaecologists (11.5%) were the main sources of information. Almost 70% of the participants correctly indicated that a Pap smear can detect cervical cancer (68.6%). When asked how often a Pap smear should be done, 65.4% of the participants indicated that this should be done once every year. About 12.4% and 6.4% revealed that this should be conducted once every three years and once every five years, respectively. More than half of the participants correctly indicated that sexually active (64.7%) women, who are 21 years and above (56.9%) or 30 years and above (55.1%) should have a Pap smear.

**Table 4:** knowledge of a Pap smear test among study participants (N=283)

Variables and categories	Frequency (n)	Percentage (%)
**Ever heard of a Pap smear test**		
Yes	269	95.1
No	6	2.1
Not specified	8	2.8
**Sources of information about a Pap smear test (n=269)**		
Nurse	127	47.2
Teacher in school	47	17.5
Gynaecologist	31	11.5
Relatives	23	8.6
Mass media	21	7.8
Friends	13	4.8
Family physician	7	2.6
**Can a Pap smear test detect cervical cancer?**		
Yes	194	68.6
No	68	24.0
Do not know	21	7.4
**How often should a Pap smear be done?**		
Once every year	185	65.4
Once every three years	35	12.4
Once every five years	18	6.4
Once every ten years	17	6.0
Other	28	9.9
**Who should have a Pap smear test ^a^**		
Women who are sexually active	183	64.7
Women 21 years and above	161	56.9
Women 30 years and above	156	55.1
Elderly women	138	48.8
Sex workers	119	42.0

a Multiple responses to the variable, therefore % are more than 100.

HPV = Human Papillomavirus; VIA = Visual inspection with acetic acid

**Attitudes towards cervical cancer screening:** the descriptive analyses of attitudes towards cervical cancer among women are presented in Table 5. The analyses revealed that 89.9% of the participants agreed with the statement that cervical cancer is highly prevalent in SA. Similarly, 90.0% of the participants agreed that any adult woman is at risk of cervical cancer. Half (54.1%) of the participants agreed that cervical cancer cannot be transmitted from one person to another.

**Table 5:** attitudes towards cervical cancer screening among study participants (N=283)

Variables and categories	Agree (positive attitude) n (%)	Disagree (negative attitude) n (%)
Attitudes	231 (81.8)	52 (18.2)
Cervical cancer is highly prevalent and the second leading cause of deaths among women in South Africa.	254 (89.9)	29 (10.1)
Any adult woman including you can acquire cervical cancer	255 (90.0)	28 (10.0)
Cervical cancer cannot be transmitted from one person to another	153 (54.1)	130 (45.9)
Pap smear causes no harm to the client	228 (80.4)	55 (19.6)
If a Pap smear is free and causes no harm, would you screen?	263 (92.8)	20 (7.2)

**Practices towards cervical cancer screening:** the results indicate that 189 (66.8%) of participants have had a Pap smear. Of those, 50.8% indicated that they have had a Pap smear once, 27.5% twice, while 21.7% have done so three times or more.

**Correlates of good knowledge on cervical cancer:** the results on correlates of good knowledge on cervical cancer are presented in Table 6. The results showed that socio-demographics were not statistically significantly associated with knowledge of cervical cancer and cervical cancer screening. Univariable logistic regression analysis showed that the age group 25-44 years (odds ratio (OR) = 1.74 [95% confidence interval (CI) = 0.59-5.13]) were about two times more likely to be knowledgeable than the younger age group (18-24 years). Furthermore, the results indicated that married women (OR = 1.11 [95% CI = 0.45-2.74]) were one time more likely to be knowledgeable than single women. Participants who were less educated (no formal education and primary education) (OR = 1.86 [95% CI = 0.70-4.93]) were almost two times more likely to be knowledgeable than those who were more educated (Table 6). Based on the results from the univariable model, there was no statistically significant relationship (at p-value of less than 0.2), as none of the variables were associated with knowledge of cervical cancer screening. Therefore, a multivariable regression analysis could not be performed.

**Table 6:** correlates of good knowledge on cervical cancer (N=283)

Variables and categories	OR [95% CI]	p-value
**Age**		
18-24	Ref	
25-44	1.74 [0.59-5.13]	0.317
45-65	1.18 [0.27-5.12 ]	0.823
**Religion**		
Christian	1.82 [0.51-6.49]	0.353
Other religions	Ref	
**Marital status**		
Single	Ref	
Married	1.11 [0.45-2.74]	0.827
**Education**		
Less education	1.86 [0.70-4.93]	0.215
More education	Ref	
**Parity**		
No children	Ref	
1-3 children	0.50 [0.14-1.73]	0.274
4 and above	0.66 [0.13-3.34]	0.619

Note: Level of significance ≤ 0.05

Ref: Reference category

95% CI = 95% confidence internal; OR = odds ratio

## Discussion

The results of this study show that more than 90% of the participants have heard of cervical cancer. This high level of awareness in this study may potentially be attributed to the fact that the research was conducted in health facilities where there is free cervical cancer screening as well as cervical cancer screening education programmes [22]. A near similar pattern of awareness was recorded in studies conducted among women in Qatar, Johannesburg in SA, Nigeria, Nepal, and India where 85%, 78.9%, 77%, 65.7% and 65.5% of women, respectively, were aware of cervical cancer [2,26,27]. The results are in contrast with a study conducted in rural Lagos State in Nigeria where only 15% of the participants had heard of cervical cancer [28]. Similarly, less than 50% of Sudanese women were aware of cervical cancer [29]. In other studies, conducted among Iraqi immigrant women living in Malaysia as well as women attending an antenatal clinic in Abakaliki Nigeria, 57.4% and 37.6%, respectively, were aware of cervical cancer [30,31]. The main source of information about cervical cancer indicated by the participants in this study was community health workers (38.2%), followed by a doctor or nurse (23.0%). Consistent findings have been reported among women from SA (Johannesburg and Limpopo) and Nigeria, where the majority of participants recorded health care workers, doctors or nurses as their key source of information [32-34]. It is not too surprising that health care workers were the major source of information in this current study, as most of these patients attend a clinic on a frequent basis for their chronic medication.

The level of knowledge of cervical cancer and screening was very low in this study; only 28% of the study participants had adequate knowledge about cervical cancer and screening. A similar meagre percentage was recorded among Zimbabwean women where less than 40% knew about cervical cancer screening [35]. Studies conducted in SA, Nigeria, Qatar and Zimbabwe reported knowledge of cervical cancer and cervical cancer screening as ‘inadequate’, ‘low level’ and ‘deficient’ [26,32,33,36]. Contrary to these studies is a study conducted in a tertiary institution in Malaysia where knowledge was said to be adequate [37]. Knowledge of cervical cancer cannot be overemphasized among rural women considering their exceptional high risk. Therefore, awareness campaigns and programmes should focus on explaining issues and disseminating health information about cervical cancer prevention. The level of knowledge of symptoms was found to be poor in this study. Abnormal vaginal bleeding was the most mentioned symptom. These results agree with findings among women in Ruvuma, Tanzania, and a similar facility-based study conducted in women of reproductive age in India where the most mentioned symptom was vaginal bleeding [38,39].

As indicated in this study, major associated factors for developing cervical cancer stated by the participants include low immunity, early age at first sexual intercourse, and multiple sexual partners - even though established global evidence showed that persistent infection by sub-types of HPV is a central cause of cervical cancer [40]. More than half of the participants did not know that infection with HPV is a necessary cause for cervical cancer. The knowledge of symptoms and factors associated with cervical cancer is crucial in both the prevention and early detection of cervical cancer. A risk factor is any factor that alters the chance of acquiring a disease such as cancer [41]. Knowledge of symptoms and factors associated with cervical cancer was poor in this study, indicating the need for education in this area. Knowledge of cervical cancer screening methods and treatment was found to be low among participants. Of those with knowledge in this area, the majority mentioned a Pap smear, while only a small proportion mentioned HPV deoxyribonucleic acid (DNA) testing and VIA. The fact that Pap smear was the most mentioned screening method is reasonable, bearing in mind that Pap smear was invented over 70 years ago [42], and it is the screening method performed in the clinics where this study was conducted.

Other screening methods such as HPV DNA testing and VIA are more recent. VIA is a promising relatively new approach, which began in September 2005 and has been incorporated into the cervical cancer prevention services in SA and some sub-Saharan African countries [42,43]. Although HPV testing and VIA are not freely offered in the SA public health sector, several non-government organisations have been performing VIA while some private laboratories offer HPV DNA testing [44]. The majority of women (81.1%) showed a good or positive attitude towards cervical cancer screening in our study. Most studies reported similar findings of good attitude owards cervical cancer screening, South Ethiopia (65.2%), Cambodia (74%), India (76.2%), Zimbabwe (80%), Nigeria (80.4%) and Qatar (85.5%) [4,12,26,27,34,45].

Most of the participants were aware of the Pap smear test (95.1%). This finding is in line with the results of studies conducted in Malaysia, Qatar and SA, where 80.5%, 76% and 60% of participants had heard of a Pap smear test [26,30,32]. The study findings further revealed a fairly good (66.8%) uptake of a Pap smear. Some of the study participants have had a Pap smear done more than once (50.8%). In studies conducted in Johannesburg and Limpopo in SA, 80% and 96.8%, respectively, had never had a Pap smear [32,33]. The uptake of Pap smear in this study is fairly good compared to some studies conducted in SA. Increased uptake among these participants could be attributed to the burden of HIV among women in this area. A yearly test is routinely done and emphasized for HIV-infected women according to South African guidelines [24]. The low uptake of cervical cancer screening among rural women is the direct outcome of factors such as knowledge and attitudes. The study focused on rural women attending PHC clinics in a particular setting in SA. Therefore, the study cannot be generalised to women in KwaZulu-Natal and SA. However, most living conditions in rural SA are similar and the study may apply to other rural settings in SA and sub-Saharan Africa.

## Conclusion

Despite the high awareness, good attitude and fair uptake of Pap smears, inadequate knowledge of cervical cancer and cervical cancer screening is of major concern. Health professionals are therefore encouraged to focus not only on creating awareness but also on providing educational information to improve knowledge and practice of cervical cancer screening. Relevant authorities should develop strategies, policies and health promotion programmes that improve knowledge and contribute to the prevention of cervical cancer among rural women.

### What is known about this topic

- *Cervical cancer represents a major cause of morbidity and death in poor-resource settings;*
- *Sub-Saharan Africa is the most affected region of the world;*
- *Factors such as social inequality, access to preventive health care, living standards and effective screening services are put forward as the major cause of the discrepancy between poor-resource and rich-resource settings*.

### What this study adds

- *Information on knowledge and attitudes of cervical cancer screening uptake among women in rural settings;*
- *Information is required to monitor the success of the national health system´s goal of screening at least 70% of women for cervical cancer;*
- *Awareness cannot be overemphasized since it does not necessarily translate to adequate knowledge and uptake of cervical cancer screening; and compared to other rural settings in South Africa and sub-Saharan Africa, uptake of cervical cancer screening is fairly good in this rural setting where the study was conducted*.
